# Intelligent security and privacy solutions for enabling personalized telepathology

**DOI:** 10.1186/1746-1596-6-S1-S4

**Published:** 2011-03-30

**Authors:** Bernd Blobel

**Affiliations:** 1 eHealth Competence Center, University Hospital Regensburg, Franz-Josef-Strauss-Allee 11, 93053 Regensburg, Germany

## Abstract

Starting with the paradigm change of health systems towards personalized health services, the paper introduces the technical paradigms to be met for enabling ubiquitous pHealth including ePathology. The system-theoretical, architecture-centric approach to mobile, pervasive and autonomous solutions has to be based on an open component system framework such as the Generic Component Model. The crucial challenge to be met for comprehensive interoperability is multi-disciplinary knowledge representation, which must be integrated into the aforementioned framework. The approach is demonstrated for security and privacy services fundamental for any eHealth or ePathology environment.

## Introduction

For increasing quality and safety of care as well as the efficiency of care processes, health systems around the globe undergo organizational and thereby structural and functional changes. They move from organization-centric to process-controlled care paradigm, also called managed care or shared care. In the organization-centric case, just locally defined policies, process definitions, workflows, terminologies, and sometimes even technologies are sufficient to determine care. There is almost no inter-organizational communication and collaboration which is clearly organized and technically supported. For managed care, the aforementioned care environment and conditions have to be (a priori) negotiated and agreed between the different players cooperatively involved in patients care. This process of changing paradigms continues towards personalized (individualized) care, where care processes are not predefined but determined by the subject of care’s status, contextual and environmental conditions, expectations, intentions, etc., also considering methodologies for individually tailoring diagnosis and therapy. This includes predictive medicine, considering genomics, public health reference data, clinical studies’ outcome, etc. If such care is not just organizationally distributed but also delivered independently of time and location of the actors and resources involved, we move to tele-medicine (or by including public health, prevention and social care to tele-health) and its specialties like tele-pathology. This requires the inclusion of enabling technologies such as information and communication technology (ICT), specialized biomedical engineering including sensors and actuators, etc. As a result, terms like eHealth, pHealth (personalized health) or mHealth (mobile health), but also ePathology have been introduced.

## Methodologies and principles

More information about paradigm changes to enable eHealth and pHealth can be found, e.g., in [1]. Following, the basic methodologies and principles like technical paradigms to be met, the need for architecture-centric, ontology-driven approaches will be shortly introduced, thereby partially extending and partially referencing the aforementioned paper. Overcoming current terminology confusions, the author endeavors to keep definitions simple and practical, starting with the definition of architectures crucial for this paper: A system’s architecture defines its components, their functions and interrelations.

### Technical paradigms to be met for ePathology

The provision of fully distributed health services independent of time and locations requires technical paradigms enabling such approach. Overall communication and collaboration is enabled by mobile technology (or in the focus to ICT by mobile computing). Remote intervention requires pervasive technology (pervasive computing) thereby bridging the gap between all principals [2] involved (systems, persons, devices, applications, components). This also includes the subject of care. Here, sensors, actuators, nano-technology, etc., come on stage. Since every subject of care is special, individualized medicine requires individualized systems. Such solutions cannot be manually produced anymore, but have to be provided automatically. This requires autonomous, i.e., adaptive, self-organizing solutions (autonomic computing). Especially the latter two paradigms require the formal representation of all systems involved.

### A practical definition of interoperability

Interoperability describes successful collaboration between actors to achieve a certain business goal. This might be supported by involving other principals according to the aforementioned OMG definition. Interoperability requires the designation of the system in question as well as its environmental relations, the intended business objectives and the actions to be taken. Thereby, system and environment have to be observed, observation have to be interpreted and the action must be specified, performed and the outcome validated in relation to the objectives. The presented information cycle must be continued if needed. Interpretations and actions require knowledge. Supporting systems have to be interconnected thereby using appropriate technical protocols. The latter is the concern of many interoperability definitions. In summary, interoperability is first of all a matter of knowledge management and not a protocol challenges.

### The GCM framework for eSystems architectures

The first challenge for designing collaborative environments is the definition of the system in consideration as mentioned. This is a highly dynamic process, as parts of a system’s environment can be included in a more complex consideration, or parts of a system can be selected for a specialized and detailed focus. As a consequence, details of a histological image, the specimen, the pathology department, or a collaborative environment of different medical specialties involved in a service request and delivery chain can be defined as system in question and of course modified for the next process step. The system may represent different domains such as medical, legal, technical, administrative, etc., also combining them to reflect special aspects of the same system. Therefore, a hospital can be considered from a medical, administrative, legal, technical, etc., viewpoint separately to reflect certain business aspects. For the real process, the different domains are impacting the system in their combination.

For separately and flexibly enabling structural and functional as well as multi-disciplinary considerations, an appropriate abstraction level is recommended, borrowing from system theory and system engineering. This abstraction will get as more concrete as more structural and functional details of the real system are included in the investigation.

**Figure 1:**
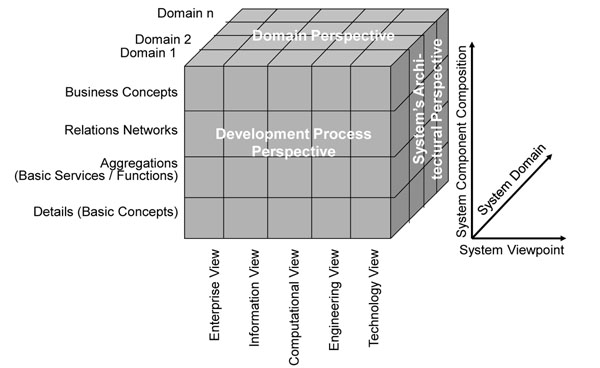
The Generic Component Model (GCM)

Many years ago already, the Generic Component Model (GCM) has been introduced, matured, and meanwhile widely deployed as abstract architectural framework for any eSystem, thereby describing the components’ composition/decomposition, the representation of the domains involved, and the ICT development process of the intended solution (Figure 1). For describing the practical process of developing ICT solutions in a model-driven way, ISO 10764 Information technology – Reference Model - Open Distributed Processing [3] and its recent updates have been used. Seeing that interoperability focuses on the business process, just the Enterprise View has to be considered. Domains describe the aforementioned aspects a system serves. Because different domains use their special language derived from a special ontology, ontologies can be deployed for separating domains.

Aggregation of components both within and between domains can just be performed at the same level of granularity! If architectural principles have been ignored as it happened for many systems such as legal, terminology or ontology ones, interrelations as well as binding to other domains’ components are poor as it can be shown in SNOMED, ICD 9 an ICD 10, etc.

## Results

### Layered security services model

**Figure 2:**
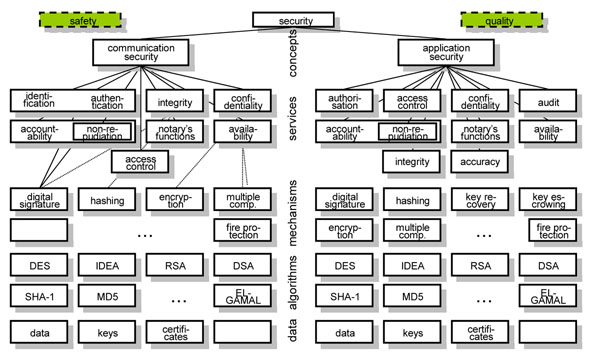
The layered security model

Security issues can be grouped into the concepts communication security and application security. Communication security deals with the connection to, and the communication between, systems, thereby preventing attacks on system and communication channel. Application security concerns the use of a system’s functions and data. Figure 2 presents the layered security services model covering the security system from concepts through services, mechanisms, algorithms and data.  Just mechanisms, algorithms and data might be specific for pathology.

**Figure 3:**
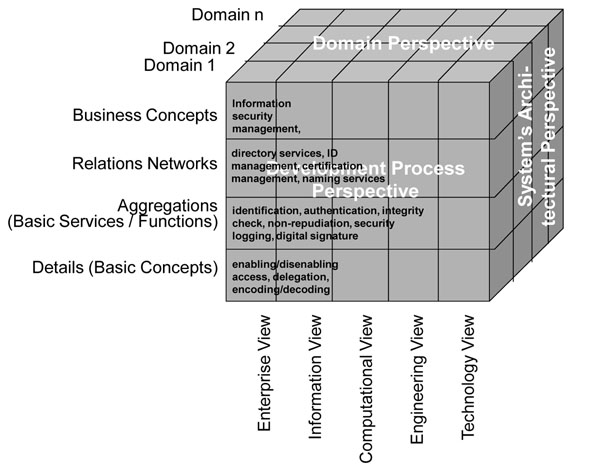
An architectural approach to the security and privacy domain

While communication security is domain-unspecific, by that way enabling the re-use of advanced communication security services developed in other domains like banking, application security is highly specific for health due to the social impact of personal health information. So, application security is the challenge we have to deal with, covering safety, security and privacy of health-related services and personal health information. While technical specifications such as the identification and authentication of entities are manageable depending on the distinguishing features used such as knowledge, tokens, properties, privacy-related services such as privilege management, authorization, access control, etc., summarized as policies, are defined in legislation, regulations, rules, consent statements or documents, codes of ethics, etc. They are usually not formally expressed and a matter of interpretation (so keeping myriads of lawyers busy). All aspects of an eHealth system such as the medical, legal, administrative and technical ones have to be managed (analyzed, specified, implemented and maintained) in an architecture-centric and formalized way. Therefore, they must be related to the architectural framework chosen, as presented in Figure 3 for security and privacy services using the GCM.

Thereby, the system’s components, their functions and interrelations – in other words, its architectural description – can be instantiated for any object such as documents, messages, devices, specimen, images, holograms, etc. So, integrity check for images can be provided by an image signature, using special algorithms to characterize color histograms, texture properties, global and local attributes. Authentication of origin or ownership can be provided by watermarks, frequently used for managing copyrights. Here, visible or invisible information is provided to an image in a way that is difficult to remove. Digital watermarks can be modified by, e.g., lossy compression of data, cropping an image or video, or intentionally adding noise. In this paper, we do not tackle steganography which enables the transportation of secret information hidden in an image.

**Figure 4:**
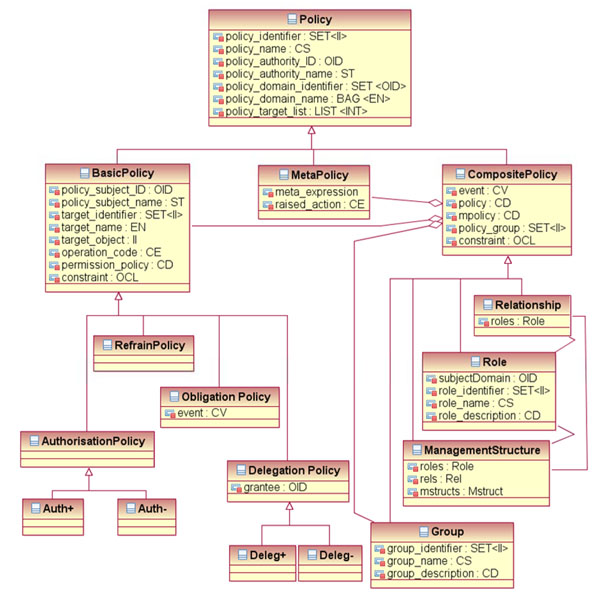
Composition/decomposition of a policy sub-domain [8]

In the next step, the architectural specification has to be developed for a security and privacy sub-domain: the domain of policies. In its general definition, a policy is a complex of legal, organizational, functional, medical, social, ethical and technical aspects, which must be considered in the context of security and privacy. A security policy defines the framework, rights and duties of principals involved, but also consequences and penalties in the case of disregarding the fixings taken (limited to persons). Using the GCM according to ISO 22600 “Health informatics – Privilege management and access control” [4], Figure 4 presents the architectural composition/decomposition of policies.

### The knowledge representation challenge

As introduced in above, interoperability is based on knowledge shared between the involved principals about the common business objectives, the business context, motivation, etc., as well as the appropriate actions taken. Therefore, knowledge representation (KR) is crucial for advanced cooperation. Since this is a difficult task within one domain already, among others depending on the complexity of a certain domain, the aforementioned challenge is even bigger in a multi-disciplinary approach like pHealth.

**Figure 5:**
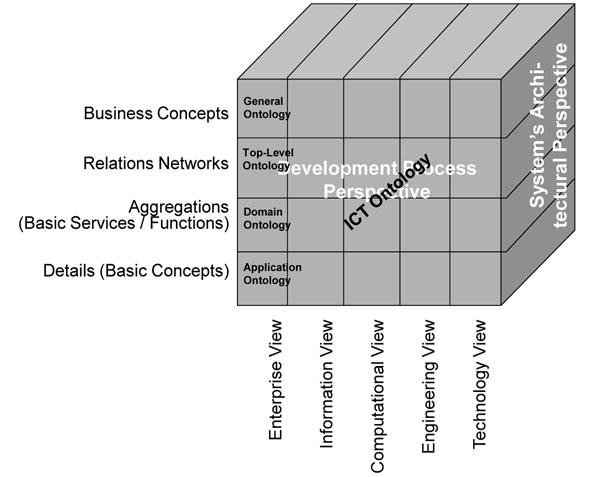
An architectural approach to ontology systems

The science dealing with the representation of structure and behavior of systems is called ontology. To avoid the endless battle between philosophers and domain experts who consider general aspects of reality vs. domain-specific specializations, a layered system of ontologies has been introduced providing an architectural approach to the system “ontology”, thereby reflecting the different granularity levels of the GCM (Figure 5).

Application ontology describes the business concepts the ICT solution should support. For achieving interoperability between different applications within a certain domain (e.g. medicine), the involved application ontologies have to be mapped using the domain ontology. Integration of different disciplines represented by different domain ontologies is moderated by top-level ontologies. Health-related ontologies contributing to an interoperability environment have been checked, harmonized, and approved as member of the Open Biomedical Ontology Foundry (OBO Foundry). They have been derived from the Basic Formal Ontology (BFO), which is a formal top-level ontology based on tested principles for ontology construction, subdividing reality into two orthogonal categories and thereby bridging the gap to the next ontology level [5]. They can be used as references to overcome weaknesses and inconsistencies of existing terminologies claiming for an ontology, and to reflect the aforementioned system of ontologies appropriately. The final criterion proofing the correctness of the approach is the universal reality (philosophically the thing itself, or the philosophical categories) independent of the scientific interpretation of special instances of the universe. So, the generalization leads from the representation of scientific concepts and their relations as knowledge representation up to the definition of universal building blocks (components) and their interrelations within the classic ontology.

Practical interoperability models describe the business case (Enterprise View) of multi-disciplinary pHealth solutions, where pathology is one domain with several sub-domains. They represent the systems in question using the corresponding application ontologies harmonized by a domain ontology. Different domains are integrated by mapping them through top-level ontologies. The resulting business model is informationally represented using ICT ontology and thereafter implemented according to a unified development process. This is, by the way, the least challenging part in the game, even if computer scientists or informaticians usually like to be the most prominent player in eSociety solutions.

The representation of both knowledge and high level ontologies is also a matter of the representation language, ranging from natural language through vocabularies, dictionaries, thesauri, meta-languages such as XML, frames, formal languages, prepositional logics, predicate logic and modal logics up to universal logic [6]. The abstraction or formalization level depends on the level of commonality achievable. As more different the domains are as more knowledge including meta-knowledge about the representation style has to be communicated, guaranteeing commonality in “understanding” just at a level of higher abstraction.

Examples for formal policies represented in XML, predicate logic, or formal languages can be found in [7].

### Privilege management and access control

Managing rights and duties of actors regarding the different personal information objects of an identified subject of care has traditionally been ignored or as much as (or even more than) possible simplified, leading to privilege or authorization attributes in databases or role-based privilege management and access control solutions in open and distributed environments. Here, actors have been grouped according to their role in a business process. Furthermore, the information objects have been classified according to their sensitivity. As a result, the relations between actors as well as between them and related objects have been dramatically reduced.

**Figure 6:**
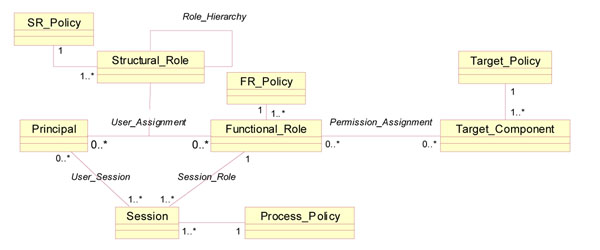
Policy-driven, role-based access control [8]

In pHealth including pPathology, such coarse-grained approach does not appropriately reflect the process context with its environmental as well as personal conditions. So, all the components have to reflect those constraints expressed by corresponding policies, as shown in Figure 6.

Structural and functional roles thereby represent the relations of a principal to an organization or to an activity within a process respectively according to ISO 21298 Health informatics – Functional and structural roles [9]. Design and specification of all architectural components of the selected business case and their binding is done highly dynamically. This concerns all domains involved including the policy domain ruling privileges and access control.

**Figure 7:**
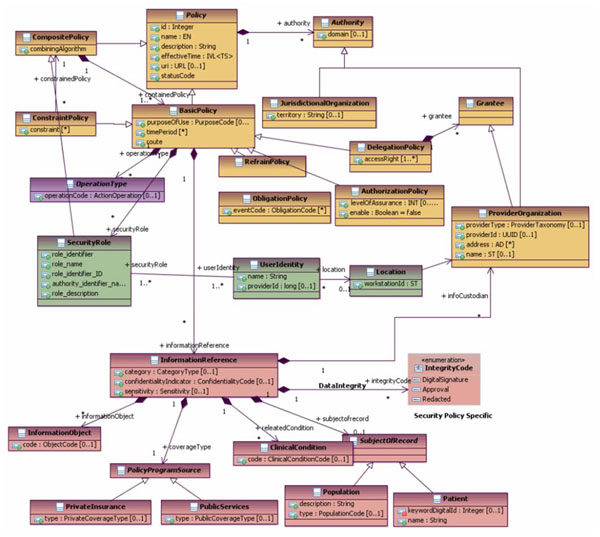
The HL7 Common Security and Privacy Domain Analysis Model [13]

A generic reference model for the informational representation of privilege management and access control in a business context has recently been standardized in the HL7 Common Security and Privacy Domain Analysis Model. This specification, provided as Draft Standard for Trial Use and thereby offering the opportunity to implement, to test and to improve the specification, is the first international standard which took up the challenge to combining the very advanced definitions of, e.g., ISO 22600, ISO 21298, OASIS SAML 1.1 [10], OASIS OASIS XACML 2.0 [11], OASIS AVDL 1.0 [12], etc., to one harmonized and comprehensive view, as demonstrated in Figure 7.

The offered model provides a combination of the composition/decomposition schema of policy base classes, of informational references (to be developed and refined in the future), the actor schema (to be developed and refined in the future) as well as the actions defined. Here, the reference to Figure 3 is recommended. The standard document also provides specifications to migrate to existing HL7 solutions like the HL7 RBAC Permission Catalogue [14], the Patient Consent Policy Document [15], and many others.

## Discussion

The presented architecture-centric approach to personalized solutions for ubiquitous care enables individualized services for caring patients, and even citizens before becoming patients, independent of time and location of actors and resources. For that purpose, a certain business case is represented as system including the relations to its environment, thereby defining the system appropriately ranging from elementary particles up to the universe. The GCM as the chosen architecture framework models a system regarding its structure and behavior in three dimensions: the different domains a system is serving, the architectural composition/decomposition of the system (to be provided for each of the considered domains), and the unified development process for analyzing, designing, specifying, implementing and deploying the ICT solution to facilitate the business process in questions. For appropriately describing the system with its domains, application ontology has to be exploited, which defines the business concepts including the terminology deployed. This application ontology has been derived from domain ontologies and is maintained and used by the domain experts. Domain ontologies enable mapping between different application ontologies within that domain, while top-level ontologies enable mapping between different domain ontologies. The system of ontologies used for the representation of the business system has to be managed separately from the system in question.

The knowledge represented in the system architecture model is contained in the concepts, which are represented by the components as well as rules to be applied to relations such as constraints for specializations or aggregations within one domain, mapping/binding of components from different domains as well as transformation within the development process. It provides the essence for successful collaboration to achieve the business objectives.

Examples for relations between the system components at different granularity level are the assignment of privileges, an authorization statement, or other processes. Beyond security and privacy, relations could represent rules such as truth statements, decision supporting rules, algorithms to evaluate parameters, by that way facilitating, e.g., (partially) automated analysis processes in pathology. An example for such knowledge-driven data representation and processing is for example given in [16] in this volume, even if the used term ontology might be problematic without the detailed considerations performed in this paper.

## Conclusion

Like any system, eHealth/pHealth systems as well as parts thereof such as tele-pathology/ePathology solutions must be analyzed, designed, specified, implemented, deployed and maintained based on a system-theoretical, engineered methodology of an architecture-centric approach. The Generic Component Model (GCM) has been proven as appropriate framework for formally describing any system in question. Meanwhile, the methodology has been validated and adopted for international standards. All architectural components defined for a pHealth business case in question are designed and implemented dynamically, thereby leading to highly flexible and personalized solutions. Aggregations of components within and between different domains are restricted to the same level of granularity, presented as neighbored components, if the relation can be logically/ontologically proven. Complexity/granularity of the system in question, the number of domains included as well as the formalization level for knowledge representation should always be limited to the level absolutely needed.

## Competing interest

The author declares that he has no competing interests.
